# Host-Specific Parasites Reveal the History and Biogeographical Contacts of Their Hosts: The Monogenea of Nearctic Cyprinoid Fishes

**DOI:** 10.3390/biology11020229

**Published:** 2022-01-31

**Authors:** Andrea Šimková, Eva Řehulková, Anindo Choudhury, Mária Seifertová

**Affiliations:** 1 Department of Botany and Zoology, Faculty of Science, Masaryk University, Kotlářská 2, 611 37 Brno, Czech Republic; evar@sci.muni.cz (E.Ř.); 108415@mail.muni.cz (M.S.); 2 Division of Natural Sciences, St. Norbert College, De Pere, WI 54115, USA; anindo.choudhury@snc.edu

**Keywords:** host-parasite associations, host-specific parasites, monogenea, fish, cyproniforms, phylogeny, biogeography, Nearctic area

## Abstract

**Simple Summary:**

Parasites exhibiting close associations with their hosts may represent a useful tool when investigating historical biogeography, especially in the case of hosts associated with a once contiguous landmass. Host-specific gill parasites (Monogenea) were applied as a supplementary tool to reveal the historical biogeographical contacts between freshwater fish from North America and Europe and their contemporary contacts in North America. Cyprinoidei is the most species-rich lineage of cypriniform fish with Leuciscidae exhibiting a Holarctic distribution. Monogenean parasites of the genus *Dactylogyrus* are mostly restricted to this freshwater fish group, and the high species diversity of *Dactylogyrus* follows the high diversity of their cyprinoid fish hosts. Using a phylogenetic approach, two Nearctic clades of *Dactylogyrus* spp. with different origins were revealed indicating two different historical routes of cyprinoid dispersion to the North American continent. Our study showed that the historical contacts between European and North American leuciscids were accompanied by the host switching of gill monogeneans. The phylogenetic relationships among North American *Dactylogyrus* spp. indicated numerous colonizations of cypriniform fish resulting from ancient paleogeographic events and contemporary drainage reorganization, thereby, facilitating contacts among phylogenetically distant fish species.

**Abstract:**

Host-specific parasites exhibit close co-evolutionary associations with their hosts. In the case of fragmented/disjunct host distribution, host-specific parasites may reflect the biogeographical history of regions and/or the role played by contacts of hosts. The present study was focused on *Dactylogyrus* (Monogenea) species almost exclusively parasitizing cyprinoid fishes. We investigated the phylogenetic relationships between *Dactylogyrus* parasites of Nearctic cyprinoids (Leuciscidae) and *Dactylogyrus* parasites of Palearctic cyprinoids and used *Dactylogyrus* phylogeny to explore the biogeography of fish hosts in Europe and North America. Phylogenetic analyses revealed that two Nearctic clades of *Dactylogyrus* spp. have different origins. Historical contacts between European and North American leuciscids were accompanied by the host switching of *Dactylogyrus* species. In the Nearctic region, *Dactylogyrus* parasites also colonized non-leuciscid fishes. *Dactylogyrus* spp. of three Nearctic leuciscid clades were included in the phylogenetic reconstruction; only *Dactylogyrus* spp. of the Plagopterinae had a common origin. *Dactylogyrus* species did not reflect the phylogenetic relationships among leuciscid clades, suggesting that past co-diversification was overshadowed by colonization events mediated by paleogeographic and climatological changes and extensive drainage reorganization. Host-specific monogeneans serve as a supplementary tool to reveal the historical biogeographical contacts between freshwater fish from the North America and Europe and also contemporary contacts of leuciscids in North America.

## 1. Introduction

Parasites are considered useful indicators of contemporary and historical ecology and biogeography on varying temporal and spatial scales [1,2]. They reveal processes involved in diversification and the formation of ecosystems and provide insights about the history and structure of faunal associations in evolutionary and ecological time [1,3,4]. The geographical distribution of parasites is limited by the distribution of their hosts (including historical and contemporary constraints on the host) and is closely associated with host dispersal capabilities. This phenomenon may be particularly evident in parasites with direct life cycles and exhibiting narrow host specificity (i.e., a parasite species is restricted to a given host species or a close range of phylogenetically-related host species).

Monogeneans are parasitic flatworms (Plathyhelminthes) with monoxenous life cycles (involving a single host), mostly infecting the gills and fins of fish. They are highly diverse in terms of species richness [5], morphology (various forms of the sclerotized parts of attachment and reproductive organs) and ecology (host and microhabitat specificities) [6,7,8,9,10]. Due to the close associations of monogeneans with their fish hosts, monogeneans represent a useful tool when investigating the historical biogeography of freshwater fish faunas with a fragmented/disjunct distribution, especially those that are associated with a once-contiguous landmass [11,12,13,14,15].

Among monogeneans, the genus *Dactylogyrus* is the most speciose, with the majority of species infecting freshwater fish of Cyprinoidei (formerly Cyprinidae, see [16]). The high species diversity of *Dactylogyrus* is closely linked to the high diversity of their cyprinoid fish hosts; however, a few non-cyprinoid hosts have also been documented [17]. Host switching (= host shifting) and parasite duplication (intrahost speciation) were revealed as the main processes of *Dactylogyrus* diversification in European cyprinoids [13,18]. 

In addition, the rapid adaptive radiation of *Dactylogyrus* in geographically isolated regions was shown to be closely associated with the diversification of their endemic cyprinoid hosts in the peri-Mediterranean area [14]. Generally, *Dactylogyrus* species have been recognized to exhibit high host specificity; a large proportion of *Dactylogyrus* species are either strict specialists (i.e., a parasite species is specific to a single host species) or intermediate specialists (i.e., a parasite species is specific to a limited range of congeneric host species) [10]. However, even among *Dactylogyrus*, some species are able to infect a wide range of phylogenetically distant and even geographically isolated host species [10,12,19,20].

Cyprinoidei is the most species-rich lineage of Cypriniformes and is currently represented by 12 families with Cyprinidae and Leuciscidae being the most diverse and widely distributed [21]. Leuciscidae (alternatively considered as Leuciscinae within Cyprinidae, see [21]) are distributed in Eurasia and North America (i.e., they exhibit a Holarctic distribution), whereas Cyprinidae (alternatively considered as Cyprininae within Cyprinidae) are found in Europe, Asia and Africa i.e., they are not native to the Nearctic region. 

Inferring from a mitogenome phylogeny, Imoto et al. [22] proposed that Leuciscidae originated in Cretaceous Europe and diverged into two phyletic groups, leuciscins and phoxinins, the latter possibly originating later in North America. However, Schönhuth et al. [21], using multiple mitochondrial and nuclear genes to infer the phylogenetic relationships within Leuciscidae, revealed the complex evolutionary history of this widespread fish group. Their study suggested multiple connections and dispersal events between Palearctic and Nearctic regions and multiple shifts of leuciscids between pelagic and benthic habitats.

The phylogeny of *Dactylogyrus* parasites has been previously applied to infer some historical biogeographical routes of cyprinoid fish and their historical contacts, such as in the Balkans [12], in Iberian Peninsula [13] and in Northwest Africa [15], all these regions exhibiting a high diversity of endemic fish fauna. These studies revealed patterns of historical cyprinoid dispersion, continental associations and coevolutionary histories that included secondary contacts and host switching. 

Benovics et al. [12] showed that, in the Balkans, the evolution of *Dactylogyrus* is associated with the historical dispersion and distribution of their cyprinoid hosts but is also affected by recent contacts between non-native and endemic cyprinoid species. Šimková et al. [15], using host-specific *Dactylogyrus* spp., supported the different origins of two Northwest African cyprinid lineages—Barbinae and Torinae—and inferred independent historical contacts between Iberian *Luciobarbus* (Barbinae) and two lineages of Northwest African cyprinids, these contacts were associated with host switches of *Dactylogyrus* parasites. 

Benovics et al. [13] suggested multiple origins of the southern European *Dactylogyrus* spp. parasitizing cyprinids of Barbinae linked with the northern route of cyprinid dispersion [23] and the southern route via Northern Africa [24]. Their study highlighted the role of continental bridges between southern Europe and North Africa playing a crucial role in the historical dispersion of cyprinids and also affecting the distribution of host-specific parasites of *Dactylogyrus*. Finally, Benovics et al. [25] revealed that the Middle East represents the area of *Dactylogyrus* diversification and suggested that the attachment organ (termed the haptor) of each *Dactylogyrus* lineage has specific morphological characteristics that are associated with a particular dispersal event proposed for cyprinids.

In view of the demonstrated fact that host-specific *Dactylogyrus* parasites represent useful indicators of biogeographical contacts among cyprinoids, we focused on *Dactylogyrus* spp. parasitizing leuciscids of the Nearctic region and the European part of the Palearctic region. Therefore, the aim of our study was to investigate the phylogenetic position of *Dactylogyrus* spp. parasitizing North American leuciscids within *Dactylogyrus* phylogeny (in particular to investigate the phylogenetic relationships between *Dactylogyrus* spp. specific to North American leuciscids and those specific to European leuciscid hosts) and to resolve the origin of North American *Dactylogyrus*. We hypothesized that phylogenetic relationships among *Dactylogyrus* spp. parasitizing Holarctic leuciscids will reflect the historical biogeography of a fish fauna divided between two continents.

## 2. Material and Methods

### 2.1. Parasite Collection

Fish hosts were collected in 2018 and 2019 from four states in the United States: Arkansas, Mississippi, New York and Wisconsin (Table 1). Specimens of *Dactylogyrus* species used in this study were extracted from the gills of freshly killed euthanized cypriniform fish and were subsequently examined using fine needles and a dissecting microscope. Cypriniform species were determined by our local collaborators (included in acknowledgements) or with the help of identification keys. One half of the monogenean (either the posterior part with haptoral sclerites or anterior part containing the male copulatory organ) was placed in a 1.5 mL Eppendorf tube with 96% ethanol for DNA extraction. 

For each monogenean species, DNA sequencing of both specimens with the ethanol-fixed posterior part and specimens with ethanol-fixed anterior part is important to avoid the misidentification of congeneric monogenean species exhibiting high similarities in attachment organ (posterior part) or in reproductive organs (anterior part). The other half of the worm was mounted on a slide and fixed with a mixture of glycerine and ammonium picrate (GAP) for species identification based on morphological characters (the sclerotized parts of the monogenean attachment organ (haptor), or the sclerotized parts of the reproductive organs—the copulatory organ and vaginal armaments). *Dactylogyrus* species were determined using available references [26,27,28,29,30,31,32,33,34,35,36,37,38,39,40].

### 2.2. DNA Extraction, Amplification and Sequencing

Ethanol preserved *Dactylogyrus* specimens were vacuum dried using a centrifugal vacuum concentrator (Eppendorf, Hamburg, Germany). Genomic DNA was extracted separately from each parasite specimen (1–10 specimens per species) using the DNeasy Blood & Tissue Kit (Qiagen, Hilden, Germany) following the manufacturer’s protocol. Two fragments of nuclear ribosomal DNA, generally considered suitable markers for monogenean species determination and widely applied in phylogenetic studies of *Dactylogyrus* [10,12,13,14,15,18,25], were analyzed as follows: (i) a fragment spanning partial 18S rDNA and internal transcribed spacer (18S rDNA + ITS1); and (ii) a fragment of partial 28S rDNA. 

The partial 18S rDNA + ITS1 fragment was amplified using the primers S1 (forward, 5′-ATTCCGATAACGAACGAGACT-3′) [41] and one of the newly designed reverse primers DactR1 (reverse, 5′-GAGCCGAGTGATCCACCACT-3′) or DactR2 (reverse, 5′-GTTCACACAGTTTGCTGCACT-3′). The second fragment, partial 28S rDNA, was amplified using primers C1 (forward, 5′-ACCCGCTGAATTTAAGCA-3′) and D2 (reverse, 5′-TGGTCCGTGTTTCAAGAC-3′) [42]. Each amplification reaction contained 1 U of Taq polymerase (Fermentas), 1× PCR buffer (Fermentas), 1.5 mM MgCl_2_, 200 µM of each dNTP, 0.5 μM (for 28S rDNA) or 0.8 μM (for the fragment including 18S rDNA and ITS1) of each primer and 5 μL of DNA extract (corresponding to 20 ng/μL). 

For DNA amplification, the following PCR conditions were used: initial denaturation at 94 °C for 2 min, 39 cycles of denaturation at 94 °C for 60 s (for 18S rDNA + ITS1) or 20 s (for 28S rDNA), annealing at 53 °C for 60 s (for 18S rDNA + ITS1) or at 56 °C for 30 s (for 28S rDNA), an extension at 72 °C for 90 s and a final extension at 72 °C for 10 min. The PCR products were checked by electrophoresis in 2% agarose gel and purified using ExoSap (Ecoli, Bratislava, Slovakia).

Sequencing was performed on an ABI 3130 DNA Genetic Analyzer (Applied Biosystems) using the BigDye Terminator v3.1 Cycle Sequencing Kit (Applied Biosystems, Foster City, CA, USA) and the same primers as those for PCR. Forward and reverse sequences were visually inspected, edited and combined into contigs using the software Sequencher (Ann Arbor, MI, USA). Newly generated sequences were deposited in GenBank (see Table 2 for accession numbers).

### 2.3. Phylogenetic Reconstruction

Phylogenetic analyses were performed using two sequence datasets. The first dataset included 31 partial 28S rDNA sequences of North American *Dactylogyrus* species obtained in the present study (only one genetic variant was included for species with intraspecific variability, i.e., *D. arcus*, *D. semotilus* and *Dactylogyrus* sp. 2, and no sequence for *D.* cf. *atromaculatus* was included in this dataset) and 37 sequences of *Dactylogyrus* species retrieved from GenBank representing four phylogenetic *Dactylogyrus* lineages revealed by Šimková et al. [15] (see Table 2 for accession numbers). Two species of Dactylogyridae, *Aliatrema cribbi* (acc. no. AY820612) and *Euryhaliotrematoides pirulum* (acc. no. AY820618), were selected as the outgroup. 

The second dataset was based on concatenated data of partial 18S rDNA + ITS1 and 28S rDNA and included 36 sequences of North American *Dactylogyrus* species (all genetic variants for North American *Dactylogyrus* species exhibiting intraspecific variability were included) and the sequences of 17 selected *Dactylogyrus* species parasitizing European fishes of Leuciscidae and Cyprinidae [15]. Mid-point rooting was applied because of the ambiguous positions of lineages II and lineages III in relation to lineage IV revealed by previous phylogenetic studies [15,18,25]. Sequence divergence for the North American species exhibiting intraspecific variability between different localities was estimated in MEGA X [43] using the p-distance model.

Phylogenetic analyses were conducted using the Maximum Likelihood (ML) and Bayesian Inference (BI) methods. Sequence alignments were performed separately for each gene in MAFFT v. 7 https://mafft.cbrc.jp/ (accessed on 15 May 2021) [44] with the G-INS-i algorithm. Gaps, hypervariable regions and ambiguously aligned regions were removed from the alignments using GBlocks v. 0.91b http://phylogeny.lirmm.fr/phylo_cgi/one_task.cgi?task_type=gblocks (accessed on 20 May 2021) [45]. Model selection was performed for each alignment partition, and the following models were selected using the Bayesian information criterion (BIC) in jModelTest v. 2.1.10 https://github.com/ddarriba/jmodeltest2 (accessed on 20 May 2021) [46]: the 28S rDNA dataset: GTR + I + G; the concatenated dataset: TPM3 + I + G for 18S rDNA, SYM + I + G for ITS1 and GTR + I + G for 28S rDNA. 

ML phylogenetic reconstruction was performed using the IQ-TREE v. 1.6.12 [47] on the W-IQ-TREE webserver (http://iqtree.cibiv.univie.ac.at (accessed on 20 May 2021)) [48]. Branch support was estimated using ultrafast bootstrap approximation [49] with 10,000 replicates. BI analyses were conducted using MrBayes v. 3.2.1 https://nbisweden.github.io/MrBayes (accessed on 30 May 2021) [50]. Four simultaneous chains (one cold and three heated) of the Markov Chain Monte Carlo (MCMC) algorithm were run twice for 10^7^ generations. Tree topologies were sampled every 100 generations, whereby the first 30% of trees from each run were discarded as “burn-in” to obtain the consensus tree and posterior probability values (PP). The convergence (where the average standard deviation of the split frequencies was lower than 0.01) and effective sampling sizes of all parameters were checked in Tracer v. 1.7.1 https://github.com/beast-dev/tracer/releases/latest (accessed on 5 June 2021) [51]. Trees were visualized and edited using FigTree v. 1.4.4 http://tree.bio.ed.ac.uk/software/figtree (accessed on 15 June 2021) [52].

Character states were mapped onto the phylogenetic trees as follows: First, states representing the character reflecting the geographical distribution of cypriniform species, and states representing different cyprinoid lineages (cyprinoid families) and the catostomid lineage were mapped onto the *Dactylogyrus* phylogeny that included all *Dactylogyrus* species analyzed. 

Next, character states representing the clades of Leuciscidae (subfamilies within Leuciscidae, following Schönhuth et al. [21]) and cypriniform lineages—Catostomidae and Cyprinidae were mapped onto the phylogenetic reconstruction that included the data set of *Dactylogyrus* species of Leuciscidae, three *Dactylogyrus* species of Catostomidae and a few *Dactylogyrus* species of Cyprinidae previously shown to be nested within the *Dactylogyrus* of European Leuciscidae [15]. The mapping was performed in Mesquite v. 3.2 https://www.mesquiteproject.org/ (accessed on 15 Jun 2021) [53].

## 3. Results

### 3.1. Dactylogyrus Species of Nearctic Cypriniform Fish

A total of 32 *Dactylogyrus* species from a total of 18 cypriniform host species (16 species of Leuciscidae and two species of Catostomidae) were recognized on the basis of morphological features (Table 2). For four *Dactylogyrus* species, multiple genetic variants were found. More specifically, two genetic variants were found for *D. arcus* (with the following *p*-distances: 0.4% for 18S rDNA, 1% for ITS1 and 0.1% for 28S rDNA) and *D. semotilus* (with *p*-distance = 0.1% for 28S rDNA), each of them parasitizing two *Luxilus* species—one from a northern locality (Wisconsin) and another from a southern locality (Arkansas). 

Two genetic variants were also found for *Dactylogyrus* sp. 2 parasitizing *Cyprinella whipplei* and *C. venusta* from two southern localities (Arkansas and Mississippi) (with the following *p*-distances: 0.1% for 28S rDNA and 0.5% for ITS1), and two genetic variants were found for *D.* cf. *atromaculatus* parasitizing *Semotilus atromaculatus* from a southern locality (Arkansas) and *Pimephales notatus* collected in a northern locality (New York) (with *p*-distance = 2.1% for ITS1). 

The richness of *Dactylogyrus* species on the fish investigated was very low; eight fish species were parasitized by a single *Dactylogyrus* species, and five fish species were parasitized by two *Dactylogyrus* species. Five fish species were parasitized by more than two *Dactylogyrus* species, i.e., from three to five *Dactylogyrus* species per fish species with *Luxilus chrysocephalus* and *P. notatus* exhibiting higher *Dactylogyrus* diversity in our sample (Table 1). Different *Dactylogyrus* species were found on the same host species collected from different localities (*C. venusta, L. chrysocephalus*, *P. notatus* and *S. atromaculatus*). *Dactylogyrus* species also exhibited high host specificity, i.e., except for four *Dactylogyrus* species with intraspecific variability, each *Dactylogyrus* species was strictly host specific (i.e., parasitizing a single host species).

### 3.2. Phylogenetic Position of Neartic Dactylogyrus Species within the Dactylogyrus Phylogeny

**Figure 1:**
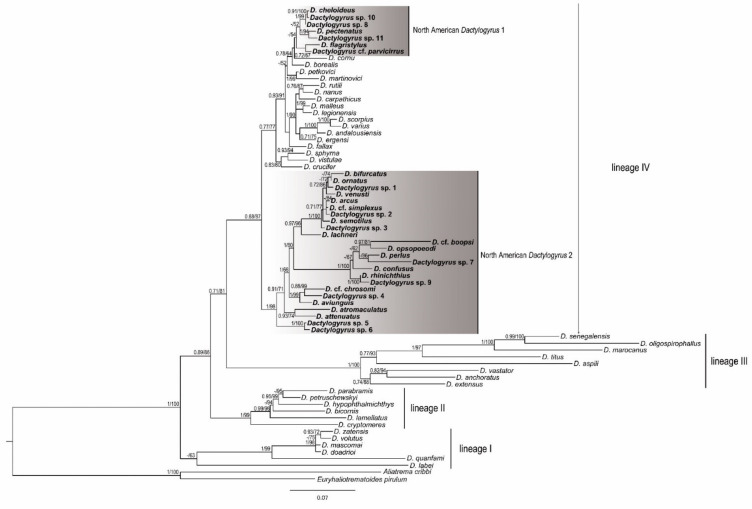
BI tree inferred from the analyses of partial 28S rDNA sequences of *Dactylogyrus* species. Numbers along the branches indicate posterior probabilities and bootstrap values resulting from BI and ML analyses. Only values >0.70 for BI and >50% for ML are shown. New sequences generated in the present study are shown in bold.

ML and BI analyses based on aligned partial 28S rDNA sequences yielded phylogenetic trees with mostly similar branching topologies and congruent nodal support values (Figure 1). Essentially, four *Dactylogyrus* lineages were recognized (lineages I–IV). The first was a well-supported lineage included species parasitizing Asian Cyprinidae (representative of Labeoninae) and a monophyletic group of *Dactylogyrus* spp. parasitizing fishes of the African Torinae and Iberian Barbinae (both Cyprinidae). However, the position of *D. labei* on Asian Labeoninae within lineage I was not supported. 

Lineage I was sister to a clade of *Dactylogyrus* spp. including lineages II, III and IV. Lineage II represented *Dactylogyrus* species parasitizing fish species belonging to Gobionidae, Xenocyprinidae and Acheilognathidae. Lineage III was formed by *Dactylogyrus* species parasitizing fish species of Cyprinidae—more specifically, Cyprininae with a Euro-Asian distribution and likely of Asian origin and African Labeoninae. Lineage IV was a large lineage that included *Dactylogyrus* species distributed on Nearctic Leuciscidae as well as three *Dactylogyrus* species parasitizing two species of Catostomidae (North American *Dactylogyrus* species were included in two clades, see Figure 1), *Dactylogyrus* species restricted to Palearctic Leuciscidae (in our phylogenetic analyses, restricted to European samples) and some *Dactylogyrus* species on Cyprinidae (European and North African Barbinae).

**Figure 2:**
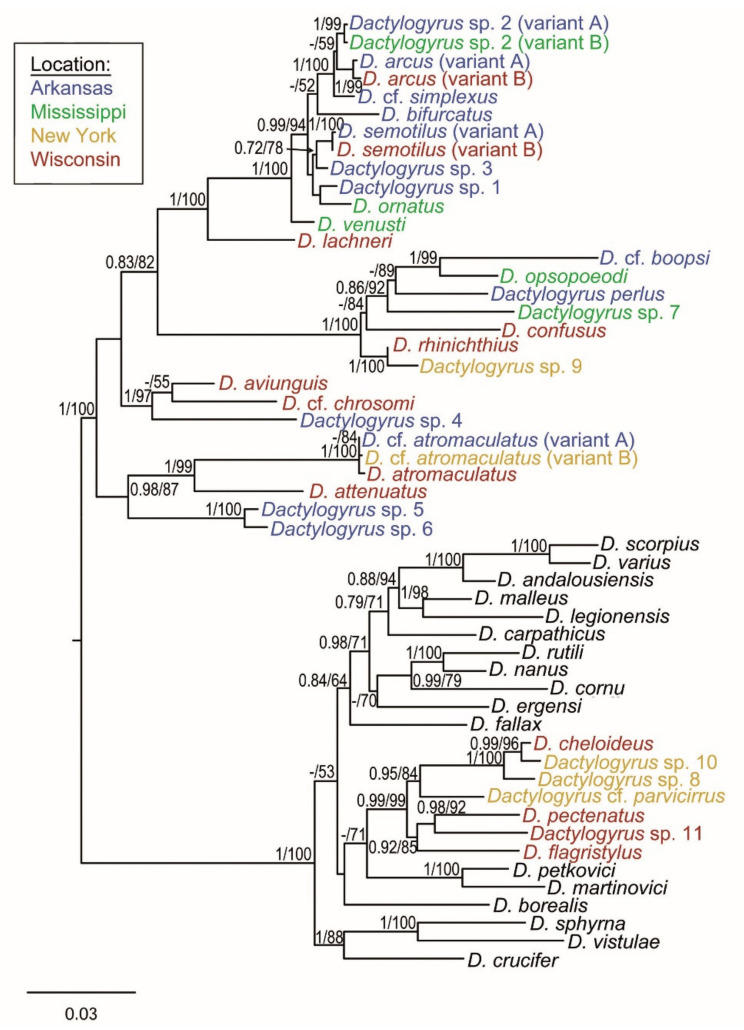
BI tree inferred from analyses of concatenated 18S rRNA, ITS1 and 28S rDNA sequences of *Dactylogyrus* species of lineage IV. Numbers along the branches indicate posterior probabilities and bootstrap values resulting from BI and ML analyses. Only values >0.70 for BI and >50% for ML are shown. Localities of collections for North American *Dactylogyrus* species are highlighted by different colors.

ML and BI analyses based on the alignment of concatenated data including partial 28S rDNA, partial 18S rDNA and ITS1 of *Dactylogyrus* species belonging to lineage IV yielded phylogenetic trees with similar branching topologies and congruent support values (Figure 2). Two clades were clearly recognized. One included strictly Nearctic *Dactylogyrus* and was formed by four well-supported subgroups, whereas the other included Nearctic *Dactylogyrus* and Palearctic *Dactylogyrus* parasitizing European Leuciscidae and Cyprinidae (Barbinae). 

In this clade, a monophyletic group of exclusively Nearctic *Dactylogyrus* was nested within Palearctic *Dactylogyrus*; however, the phylogenetic relationships between this Nearctic *Dactylogyrus* group and two European species *D. petkovici* and *D. martinovici* parasitizing Balkan endemic leuciscids was only weakly supported by ML analysis, and the phylogenetic position of *D. borealis* parasitizing *Phoxinus phoxinus* was not supported.

### 3.3. Origin of Nearctic Dactylogyrus

**Figure 3:**
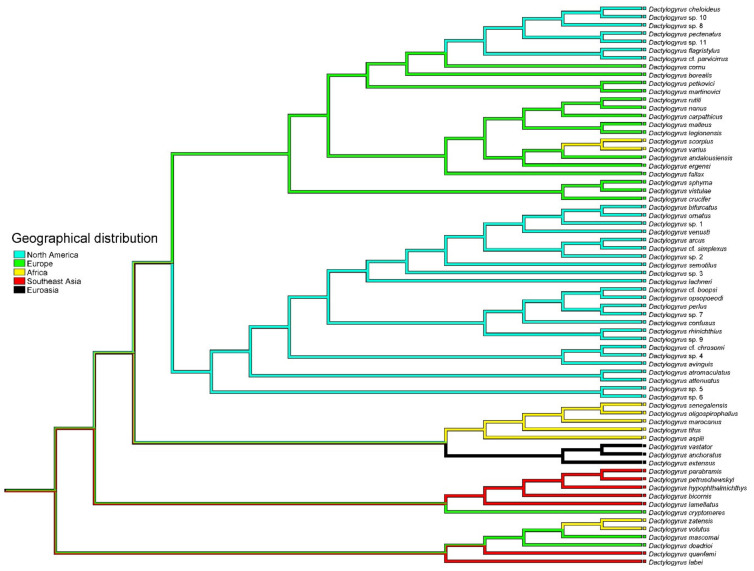
Mapping of the geographical distribution of fish hosts onto the BI reconstruction of *Dactylogyrus* phylogeny.

**Figure 4:**
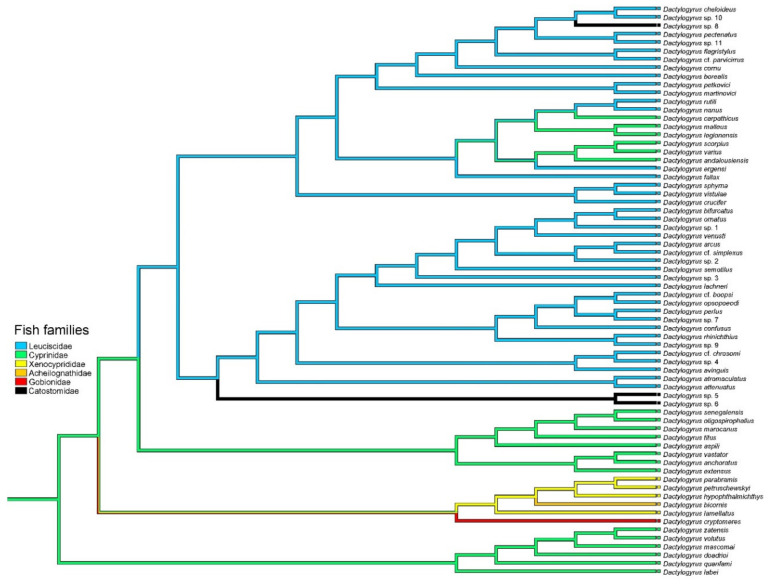
Mapping of fish lineages (of the cyprinoid families defined by Schönhuth et al. (2018) and Catostomidae)) onto the BI reconstruction of *Dactylogyrus* phylogeny.

The mapping of the geographical distribution of fish hosts onto the phylogeny of all four *Dactylogyrus* lineages (Figure 3) clearly indicated two independent origins of Nearctic *Dactylogyrus*. North American *Dactylogyrus* 1 (as defined in Figure 1) originated from Europe, whilst the origin of North American *Dactylogyrus* 2 was not fully resolved on the basis of the area mapped. The mapping of fish families (cyprinoid families and Catostomidae) onto the phylogeny of all four *Dactylogyrus* lineages (Figure 4) showed that Leuciscidae were colonized by *Dactylogyrus* from Cyprinidae, and Catostomidae were colonized twice from Leuciscidae.

**Figure 5:**
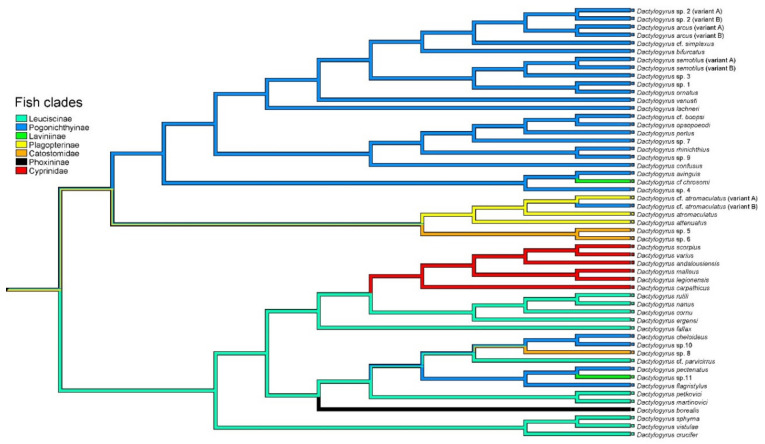
Mapping of fish clades (Cyprinidae, Catostomidae and the subfamilies of Leuciscidae) onto the BI phylogenetic reconstruction of *Dactylogyrus* of lineage IV. Clades of Leuciscidae (defined by Schönhuth et al. (2018)) are represented by Pogonichthyinae (NA clade), Leuciscinae (OW clade), Laviniinae (WC clade), Plagopterinae (CCP clade) and Phoxininae (PHX clade).

The mapping of leuciscid clades (i.e., at the level of subfamilies) defined by Schönhuth et al. (2018) onto lineage IV of *Dactylogyrus* (Figure 5) clearly indicated that the OW clade (Leuciscinae) is an ancestral host group for *Dactylogyrus* of the group, including North American clade 1. Within North American *Dactylogyrus* 1, colonization from the OW clade (Leuciscinae) to the NA clade (Pogonichthyinae) was evidenced. Moreover, our mapping revealed two independent colonisations of the WC clade (Laviniinae represented by *Chrosomus neogaeus* in our data) by *Dactylogyrus* parasitizing fish of the NA clade. The origin of *Dactylogyrus* cf. *parvicirrus* parasitizing a single Nearctic representative of the OW clade (*Notemigonus crysoleucas*) was unclear—either this species was colonized by *Dactylogyrus* from European Leuciscinae or from Nearctic Pogonichthyinae (NA clade).

*Dactylogyrus* species parasitizing Catostomidae have multiple origins. Two sister species parasitizing *Hypentelium nigricans* shared a common origin with *Dactylogyrus* spp. parasitizing fish of the CCP clade (Plagopterinae represented by *S. atromaculatus* in our data), whilst *Dactylogyrus* sp. 8 shared a common origin with two sister species parasitizing *Rhinichthys* (Pogonichthyinae). A host switch by *D.* cf. *atromaculatus* from *S. atromaculatus* (Plagopterinae) to *P. notatus* (Pogonichthyinae) was also revealed.

## 4. Discussion

The present study was focused on monogeneans of the genus *Dactylogyrus* restricted almost exclusively to cyprinoid fish species, with a view to using host-specific parasites as a potential tool for inferring biogeographical contacts between freshwater fish with fragmented distributions. Here, we investigated the phylogenetic position and origin of Nearctic *Dactylogyrus* spp. parasitizing Leuciscidae that also switched to a few species of Catostomidae in the North American continent. We expected the phylogenetic relationships among host-specific *Dactylogyrus* to reflect the historical relationships and contacts hypothesized between Nearctic and Palearctic cyprinoids [21,22].

Our phylogenetic reconstructions revealed that *Dactylogyrus* species parasitizing Leuciscidae in North America belong to two clades. The first clade of Nearctic *Dactylogyrus* species (i.e., North American *Dactylogyrus* clade 1 well supported on the basis of phylogenetic analyses using the concatenated data of 18SrDNA, 28SrDNA and ITS1) was restricted to north-eastern parts of the USA (Wisconsin and New York) and was nested within *Dactylogyrus* parasitizing European Leuciscidae and North-West African Cyprinidae. This finding indicates the European origin of North American *Dactylogyrus* clade 1, which is consistent with phylogenetic and biogeographical studies indicating that Leuciscidae originated in Cretaceous Europe [22]. The second clade (North American *Dactylogyrus* 2) included exclusively Nearctic *Dactylogyrus* spp. from Leuciscidae in north-eastern (Wisconsin and New York) and southern parts of the USA (Mississippi and Arkansas).

Recent phylogenetic analyses of Leuciscidae have indicated the independent evolution of leuciscin and phoxinin clades [21,22]. Imoto et al. [22] showed that the Far East Asian phoxinin (FEA) clade is closer to the North American phoxinin clade (Western clade, WC) than the European leuciscin clade (Old Word clade, OW) and proposed that phoxinins dispersed from North America to the Far East across the Bering Land Bridge in the Late Cretaceous or Paleocene [54,55]. Schönhuth et al. [21] included all clades of Holarctic Leuciscidae in their phylogenetic reconstruction and showed that Palearctic and Nearctic Leuciscidae do not form separate monophyletic groups. In contrast to the study by Imoto et al. [22], they indicated that the FEA clade, i.e., Pseudaspininae, occupied the basal position to all other clades of Leuciscidae, and that the WC clade, i.e., Laviniinae, occupied a sister position to the other Nearctic clades (i.e., the NA clade representing Pogonichthyinae and the CCP clade representing Plagopterinae) and to Palearctic leuciscids (the OW clade, i.e., Leuciscinae, which includes also a single Nearctic species *N. crysoleucas* and the Eurasian *Phoxinus* (PHX) clade, i.e., Phoxininae). 

However, the phylogenetic position of *D. borealis*, specific to *P. phoxinus*, a single representative of the PHX clade in our study, does not reflect the phylogenetic relationships among clades of Leuciscidae proposed by Imoto et al. [22] or Schönhuth et al. [21], as the phylogenetic position of *D. borealis* was not supported in our phylogenetic reconstruction (according to the host-parasite database, https://www.nhm.ac.uk/research-curation/scientific-resources/taxonomy-systematics/host-parasites/database/search.jsp (accessed on 20 August 2021), *D. borealis* is even documented in some representatives of the FEA clade). Concerning the two *Dactylogyrus* species parasitizing *C. neogaeus,* a single representative of the WC clade in our study, their phylogenetic positions did not follow the phylogenetic position of host species in the molecular phylogenetic reconstruction of Leuciscidae, i.e., the basal position of Laviniinae to other Nearctic and Palearctic clades.

On the basis of the mapping of fish geographical distribution onto the reconstruction of parasite phylogeny, different origins of the two Nearctic *Dactylogyrus* clades were supported, i.e., a European origin for North American clade 1 and likely an East Asian origin for North American clade 2. Surprisingly, four leuciscid species (from a total of 10 leuciscid species parasitized by at least two *Dactylogyrus* species) harbored *Dactylogyrus* species from the two divergent North American clades with different origins, which is in contrast to the pattern of *Dactylogyrus* speciation previously demonstrated for European *Dactylogyrus* [18], i.e., intrahost speciation. 

This seems to indicate that even some Nearctic leuciscid species were colonized independently by *Dactylogyrus* of different origins. The ancestor of North American clade 1 may have originated during the period of historical connections between Europe and North America, as indicated also for the historical dispersion of leuciscid fish. Two major North Atlantic land bridges were suggested to play a role in such connections, with the Thulean Bridge as the most important route for the exchange of biota between Europe and North America in the Early Tertiary. This land bridge connected southern Europe to eastern North America and was closed in the Early Eocene [56,57]. The second potential but less important trans-Atlantic connection for biota exchange was the northern De Greer Bridge between Scandinavia and eastern North America, which persisted until the Late Eocene. However, to clearly resolve the origin of the clade of North American *Dactylogyrus* 2 parasitizing Leuciscidae, we suggest that the additional sampling of Asian representatives of *Dactylogyrus* parasitizing Leuciscidae is necessary for future phylogenetic studies, i.e., particularly *Dactylogyrus* species parasitizing Pseudoaspininae (FEA clade).

It is generally hypothesized that cyprinoids originated in Oriental Asia, i.e., South and Southeast Asia [58,59]. Fossil records in Asia and phylogenetic analyses indicating that the FEA clade is a sister to all remaining clades of European and North American leuciscids support the hypothesis that ancestral Asian leuciscids dispersed in Eurasia and also colonized North America via the Bering Land Bridge, when the sea level decreased during the mid-Oligocene [21,60]. Therefore, an Asian origin for the clade of North American *Dactylogyrus* 2 appears to be a highly plausible scenario. This hypothesis may also be supported by the mapping of fish families onto the phylogenetic reconstruction of *Dactylogyrus* spp. in the present study, where Cyprinidae, which are of Asian origin, were shown as an early and potentially ancestral host group for *Dactylogyrus* spp., and Leuciscidae represented more recently evolved host groups for *Dactylogyrus*. A similar finding was revealed from previous reconstructions of *Dactylogyrus* phylogeny [15,18].

Different clades of Leuciscidae are distributed in Europe and North America. In Europe, two clades of Leuciscidae are represented—namely, the highly diverse Leuciscinae and the species poor Phoxininae [21], whilst in the Nearctic region, four clades of Leuciscidae are present—Laviniinae; Plagopterinae; Pogonichthiynae, which is the most diverse; and a single representative of Leuciscinae (see above). The mapping of leuciscid clades onto the phylogenetic reconstruction of *Dactylogyrus* indicates that ancestral European *Dactylogyrus* parasitizing Leuciscinae likely switched to colonize representatives of the most diverse Nearctic clade of Leuciscidae, i.e., the NA clade—Pogonichthyinae. 

A single *Dactylogyrus* species (*D.* cf. *parvicirrus*) identified on *N. crysoleucas,* the only Nearctic representative of Leuciscinae, was nested within the clade of North American *Dactylogyrus* 1, which has a European origin. The divergence between *N. crysoleucas* and other European leuciscin species was estimated to 37.1 Mya [22], when the land bridge between two continents was still present. Even though the mapping of leuciscid clades onto *Dactylogyrus* lineage IV revealed an unclear origin for *Dactylogyrus* host-specific to *N. crysoleucas*, it seems that *N. crysoleucas* likely lost its original host-specific gill monogeneans during its colonization of North America and was secondarily colonized by *Dactylogyrus* in North America (likely from Pogonichthyinae).

The mapping of leuciscid clades onto the *Dactylogyrus* phylogeny showed that highly diversified Pogonichthyinae is an evolutionarily old host group for Nearctic *Dactylogyrus*. Within North America clade 1, *Dactylogyrus* of Pogonichthyinae likely secondarily colonized *N. crysoleucas*, Laviniinae (represented by *C. neogaeus* in our analyses) and even a representative of Catostomidae (*Catostomus commersonii*). Within the clade of North American *Dactylogyrus* 2, *Dactylogyrus* of Pogonichthyinae likely secondarily colonized Plagopterinae (represented by the widely distributed *S. atromaculatus* in our analyses), Laviniinae (*C. neogaeus*) and even Catostomidae (*H. nigricans*). Considering the enormous diversity of Nearctic leuciscids and our limited knowledge on the potential diversity of their host-specific *Dactylogyrus* species (see [20]), this mapping—even if performed with a limited number of *Dactylogyrus* species—indicated that the two independent colonisations by *Dactylogyrus* from highly diversified Pogonichthyinae to Laviniinae likely occurred because of the sympatric distributions of North American leuciscids of both clades.

*Dactylogyrus* species are primarily parasites of cyprinoid fish (previously classified as cyprinids), i.e., 95% of species of *Dactylogyrus* are restricted to cyprinoid species. However, some *Dactylogyrus* species have also been reported on non-cyprinoid fish [17], including Catostomidae. Catostomidae are almost exclusively native to North America. *Catostomus catostomus* is the only extant trans-Pacific species [61], i.e., this species exhibits a disjunct distribution in the Nearctic and Palearctic (Siberia) regions, and *Myxocyprinus asiaticus* is the only catostomid species endemic to Eurasia and in eastern China [62]. 

Up to now, nine species of *Dactylogyrus* have been described from eight catostomid species of three genera in North America (*Moxostoma*, *Hypentelium* and *Thoburnia*, all representatives of the subfamily Catostominae) [20]. Our phylogenetic analyses included only three *Dactylogyrus* species collected on two catostomine species, *H. nigricans* and *C. commersonii* and revealed two independent colonization events involving *Dactylogyrus* on catostomine fish of North America. Without molecular calibration, it is impossible to estimate whether these colonization events represent deeper historical or more contemporary host switches resulting from the sympatric distribution of leuciscids and catostomids. However, catostomines diverged in the Oligocene (Catostomini and Erimyzonini) and in the early Miocene (Moxostomatini and Thoburniini), and the genus *Catostomus* diversified from 17.65 Ma ago in the early-mid Miocene [63]. The arrival of cyprinoids in North America during the Oligocene is hypothesized on the basis of fossil records of North American teleost fishes [60,64], i.e., in the period when some genera of catostomines were already present in North America.

In our phylogenetic reconstruction, one colonization of Catostomidae by *Dactylogyrus* was documented within the clade of North American *Dactylogyrus* 2. Two sister species of *Dactylogyrus* found on *H. nigricans* formed a monophyletic group with *Dactylogyrus* species parasitizing *S. atromaculatus* (a representative of North American Plagopterinae). We could hypothesize a host switch by *Dactylogyrus* from *S. atromaculatus* to *H. nigricans*, which may be supported by the fact that *S. atromaculatus* is one of the most common fish species in eastern North America and also widely introduced across North America [65]. 

The northern *H. nigricans* is native to southern Canada and the eastern and southern United States. It lives in the rivers of the Mississippi Basin, its range extending from Oklahoma and Alabama northward to Minnesota [66]. The sympatric distribution of both species was also documented in our study. The other host switch by *Dactylogyrus* from Leuciscidae to Catostomidae was revealed within North American clade 1. *Dactylogyrus* sp. 8 parasitizing *C. commersonii* formed a well-supported clade with two *Dactylogyrus* species parasitizing species of *Rhinichthys* (representatives of the highly diversified Pogonichthyinae), which clearly provides further evidence of another host switch by *Dactylogyrus* to catostomids on the North American continent.

## 5. Conclusions

Host-specific monogeneans of the genus *Dactylogyrus* parasitizing Nearctic cypriniform fish (including Leuciscidae and a few species of Catostomidae) formed two independent clades with different origins likely associated with different historical routes of cyprinoid dispersion to the North American continent. The relationships among *Dactylogyrus* parasitizing different leuciscid clades in North America and Europe did not reflect the phylogenetic relationships of their leuciscid hosts. 

However, the phylogenetic reconstruction of *Dactylogyrus* reflects the biogeographical contacts of cyprinoids across continents and even more contemporary contacts among leuciscids in the Nearctic region following the separation of the continents. We highlight the usefulness of host-specific monogeneans as a tool for studying the biogeographical contacts of hosts with fragmented/disjunct distributions.

## Figures and Tables

**Table 1:** List of cypriniform species collected from North America, including sites of collection, fish sample size and list of identified *Dactylogyrus* species.

Fish Species	Country	Locality	Body Water	Sample Size	*Dactylogyrus* Species
*Campostoma spadiceum* (Girard, 1856)	Arkansas	Polk County	Bear Creek	11	*Dactylogyrus* sp. 4
*Catostomus commersonii* (Lacepède, 1803)	New York	Cooperstown	Oaks Creek	12	*Dactylogyrus* sp. 8
*Chrosomus neogaeus* (Cope, 1867)	Wisconsin	Door County	Mink River	13	*D*. cf. *chrosomi*, *Dactylogyrus* sp. 11
*Clinostomus elongatus* (Kirtland, 1840)	Wisconsin	Brown County	Baird Creek, Green Bay	11	*D. confusus*
*Cyprinella venusta* Girard, 1856	Mississippi	Oxbow south of Cumbest Bridge landing	Pascagoula River	13	*D. venusti*
	Mississippi	Moon Lake	Pascagoula River	12	*Dactylogyrus* sp. 7, *Dactylogyrus* sp. 2 variant B
*Cyprinella whipplei* Girard, 1856	Arkansas	Polk County	Caddo River	2	*Dactylogyrus* sp. 2 variant A
*Hypentelium nigricans* (Lesueur, 1817)	Arkansas	Montgomery County	Huddleston Creek	3	*Dactylogyrus* sp. 5, *Dactylogyrus* sp. 6
*Luxilus chrysocephalus* Rafinesque, 1820	Arkansas	Polk County	Caddo River	3	*D. arcus* variant A, *D. semotilus* variant A, *Dactylogyrus* sp. 1, *Dactylogyrus* sp. 3
	Arkansas	Polk County	Big Fork Creek	3	*D. perlus*
*Luxilus cornutus* (Mitchill, 1817)	Wisconsin	Brown County	West Twin River	10	*D. arcus* variant B, *D. semotilus* variant B
*Nocomis biguttatus* (Kirtland, 1840)	Wisconsin	Brown County	West Twin River	7	*D. avinguis*, *D. flagristylus*, *D. lachneri*
*Notemigonus crysoleucas* (Mitchill, 1814)	New York	Cooperstown	Rom Hill Beaver Pond	5	*D.* cf. *parvicirrus*
*Notropis petersoni* Fowler, 1942	Mississippi		Bluff Creek	2	*D. ornatus*
*Opsopoeodus emiliae* Hay, 1881	Mississippi		Bluff Creek	1	*D. opsopoeodi*
*Pimephales notatus* (Rafinesque, 1820)	New York	Cooperstown	Leatherstocking Creek	4	*D.* cf. *atromaculatus* variant B
	Arkansas	Polk County	Bear Creek	2	*D. bifurcatus*, *D.* cf. *boopsi*, *D.* cf. *simplexus*
*Pimephales promelas* Rafinesque, 1820	Wisconsin	Door County	Hickory Oak Pond	7	*D. pectenatus*
*Rhinichthys atratulus* (Hermann, 1804)	Wisconsin	Brown County	Baird Creek, Green Bay	2	*D. cheloideus*, *D. rhinichthius*
*Rhinichthys cataractae* (Valenciennes, 1842)	New York	Cooperstown	Leatherstocking Creek	5	*Dactylogyrus* sp. 9, *Dactylogyrus* sp. 10
*Semotilus atromaculatus* (Mitchill, 1818)	Arkansas	Polk County	Big Fork Creek	7	*D.* cf. *atromaculatus* variant A
	Wisconsin	Brown County	Baird Creek, Green Bay	16	*D. atromaculatus*, *D. attenuatus*

**Table 2:** List of *Dactylogyrus* species used in phylogenetic analyses, their fish host species, country of collection and GenBank accession numbers for DNA sequences.

Dactylogyrus Species	Cypriniform Host Species	Cypriniform Family	Cypriniform Subfamily	Sampling Locality	GenBank Accession No.	
					28S rDNA	18S rDNA and ITS1
Lineage I						
*D. doadrioi* El Gharbi, Renaud & Lambert, 1993	*Luciobarbus guiraonis* (Steindachner, 1866)	Cyprinidae	Barbinae	Spain	KY629346	—
*D. labei* Musselius & Gusev, in Gusev, 1976	*Gibelion catla* (Hamilton, 1822)	Cyprinidae	Labeoninae	India	JX566720	—
*D. mascomai* El Gharbi, Renaud & Lambert, 1992	*Luciobarbus guiraonis* (Steindachner, 1866)	Cyprinidae	Barbinae	Spain	KY629348	—
*D. quanfami* Ha Ky, 1971	*Cirrhinus molitorella* (Valenciennes, 1844)	Cyprinidae	Labeoninae	China	EF100536	—
*D. volutus* El Gharbi, Birgi & Lambert, 1994	*Carasobarbus fritschii* (Günther, 1874)	Cyprinidae	Torinae	Morocco	KY629353	—
*D. zatensis* El Gharbi, Birgi & Lambert, 1994	*Carasobarbus fritschii*	Cyprinidae	Torinae	Morocco	KY629352	—
Lineage II						
*D. bicornis* Malewitzkaja, 1941	*Rhodeus meridionalis* Karaman, 1924	Acheilognathidae	—	Greece	KY629345	—
*D. cryptomeres* Bychowsky, 1934	*Gobio gobio* (Linnaeus, 1758)	Gobionidae	—	Czech Republic	AJ969947	—
*D. hypophthalmichthys* Akhmerov, 1952	*Hypophthalmichthys molitrix* (Valenciennes, 1844)	Xenocyprididae	—	China	EF100532	—
*D. lamellatus* Akhmerow, 1952	*Ctenopharyngodon idella* (Valenciennes, 1844)	Xenocyprididae	—	China	AY307019	—
*D. parabramis* Akhmerov, 1952	*Megalobrama terminalis* (Richardson, 1846)	Xenocyprididae	—	China	EF100534	—
*D. petruschewskyi* Gusev, 1955	*Megalobrama amblycephala* Yih, 1955	Xenocyprididae	—	China	AY548927	—
Lineage III						
*D. anchoratus* (Dujardin, 1845) Wagener, 1857	*Carassius gibelio* (Bloch, 1782)	Cyprinidae	Cyprininae	Croatia	KY863555	—
*D. aspili* Birgi & Lambert, 1987	*Enteromius macrops* (Boulenger, 1911)	Cyprinidae	Smiliogastrinae	Senegal	KY629359	—
*D. extensus* Mueller & Van Cleave, 1932	*Cyprinus carpio* Linnaeus, 1758	Cyprinidae	Cyprininae	Czech Republic	AJ969944	—
*D. marocanus* El Gharbi, Birgi & Lambert, 1994	*Labeobarbus maroccanus* (Günther, 1902)	Cyprininae	Torinae	Morocco	MW218579	—
*D. oligospirophallus* Paperna, 1973	*Labeo coubie* Rüppell, 1832	Cyprinidae	Labeoninae	Senegal	KY629361	—
*D. senegalensis* Paperna, 1969	*Labeo senegalensis* Valenciennes, 1842	Cyprinidae	Labeoninae	Senegal	KY629363	—
*D. titus* Guegan, Lambert & Euzet, 1988	*Labeo senegalensis*	Cyprinidae	Labeoninae	Senegal	KY629364	—
*D. vastator* Nybelin, 1924	*Carassius gibelio* (Bloch, 1782)	Cyprinidae	Cyprininae	Czech Republic	KY629366	—
Lineage IV						
*Dactylogyrus andalousiensis* El Gharbi, Renaud & Lambert, 1993	*Luciobarbus sclateri* (Günther, 1868)	Cyprinidae	Barbinae	Portugal	KY629351	KY629331
*Dactylogyrus borealis* Nybelin, 1937	*Phoxinus bigerri* Kottelat, 2007	Leuciscidae	Phoxininae	Spain	MN338222	MN365688
*Dactylogyrus carpathicus* Zakhvatkin, 1951	*Barbus barbus* (Linnaeus, 1758)	Cyprinidae	Barbinae	Czech Republic	KY201111	KY201098
*Dactylogyrus cornu* Linstow, 1878	*Vimba vimba* (Linnaeus, 1758)	Leuciscidae	Leuciscinae	Czech Republic	KY629371	KY629342
*Dactylogyrus crucifer* Wagener, 1857	*Rutilus rutilus* (Linnaeus, 1758)	Leuciscidae	Leuciscinae	Czech Republic	KY629374	AJ564120
*Dactylogyrus ergensi* Molnár, 1964	*Chondrostoma nasus* (Linnaeus, 1758)	Leuciscidae	Leuciscinae	Greece	MG792989	MG792874
*Dactylogyrus fallax* Wagener, 1857	*Vimba vimba*	Leuciscidae	Leuciscinae	Czech Republic	KY629370	KY629341
*Dactylogyrus legionensis* Gonzalez Lanza & Alvarez Pellitero, 1982	*Luciobarbus guiraonis* (Steindachner, 1866)	Cyprinidae	Barbinae	Spain	KY629350	KY629330
*Dactylogyrus malleus* Linstow, 1877	*Barbus barbus*	Cyprinidae	Barbinae	Czech Republic	KY201112	KY201099
*Dactylogyrus martinovici* Ergens, 1970	*Pachychilon pictum* (Heckel & Kner, 1858)	Leuciscidae	Leuciscinae	Albania	MG793000	MG792884
*Dactylogyrus nanus* Dogiel & Bychowsky, 1934	*Rutilus rutilus*	Leuciscidae	Leuciscinae	Czech Republic	AJ969942	AJ564145
*Dactylogyrus petkovici* Ergens, 1970	*Pachychilon pictum*	Leuciscidae	Leuciscinae	Albania	MG793002	MG792886
*Dactylogyrus rutili* Gloser, 1965	*Leucos basak* Heckel, 1843	Leuciscidae	Leuciscinae	Albania	MG793020	MG792904
*Dactylogyrus scorpius* Rahmouni, Řehulková & Šimková, 2017	*Luciobarbus rifensis* Doadrio, Casal-Lopez & Yahyaoui, 2015	Cyprinidae	Barbinae	Morocco	KX553860	KX578023
*Dactylogyrus sphyrna* Linstow, 1878	*Rutilus rutilus*	Leuciscidae	Leuciscinae	Czech Republic	AJ969943	AJ564154
*Dactylogyrus varius* Rahmouni, Řehulková & Šimková, 2017	*Luciobarbus maghrebensis* Doadrio, Perea & Yahyaoui, 2015	Cyprinidae	Barbinae	Morocco	KX553863	KX578026
*Dactylogyrus vistulae* Prost, 1957	*Squalius prespensis* (Fowler, 1977)	Leuciscidae	Leuciscinae	Albania	KY629369	KY629340
*D. arcus* Rogers, 1967 (variant A)	*Luxilus chrysocephalus* Rafinesque, 1820	Leuciscidae	Pogonichthyinae	Arkansas	OM108517	OM108553
*D. arcus* Rogers, 1967 (variant B)	*Luxilus* sp.	Leuciscidae	Pogonichthyinae	Wisconsin	OM108518	OM108554
*D. atromaculatus* Mizelle, 1938	*Semotilus atromaculatus*	Leuciscidae	Plagopterinae	Wisconsin	OM108519	OM108555
*D.* cf. *atromaculatus* Mizelle, 1938 (variant A)	*Semotilus atromaculatus* (Mitchill, 1818)	Leuciscidae	Plagopterinae	Arkansas	OM108523	OM108559
*D.* cf. *atromaculatus* Mizelle, 1938 (variant B)	*Pimephales notatus* (Rafinesque, 1820)	Leuciscidae	Pogonichthyinae	New York	OM108524	OM108560
*D. attenuatus* Mizelle & Klucka, 1953	*Semotilus atromaculatus*	Leuciscidae	Plagopterinae	Wisconsin	OM108520	OM108556
*D. aviunguis* Chien, 1974	*Nocomis biguttatus* (Kirtland, 1840)	Leuciscidae	Pogonichthyinae	Wisconsin	OM108521	OM108557
*D. bifurcatus* Mizelle, 1937	*Pimephales notatus*	Leuciscidae	Pogonichthyinae	Arkansas	OM108522	OM108558
*D.* cf. *boopsi* Cloutman, 1994	*Pimephales notatus*	Leuciscidae	Pogonichthyinae	Arkansas	OM108525	OM108561
*D. cheloideus* Rogers, 1967	*Rhinichthys atratulus* (Hermann, 1804)	Leuciscidae	Pogonichthyinae	Wisconsin	OM108531	OM108567
*D.* cf. *chrosomi* Hanek, Molnár & Fernando, 1975	*Chrosomus neogaeus* (Cope, 1867)	Leuciscidae	Laviniinae	Wisconsin	OM108526	OM108562
*D. confusus* Mueller, 1938	*Clinostomus elongatus* (Kirtland, 1840)	Leuciscidae	Pogonichthyinae	Wisconsin	OM108529	OM108565
*D. flagristylus* Chien, 1974	*Nocomis biguttatus*	Leuciscidae	Pogonichthyinae	Wisconsin	OM108530	OM108566
*D. lachneri* Chien, 1971	*Nocomis biguttatus*	Leuciscidae	Pogonichthyinae	Wisconsin	OM108532	OM108568
*D. opsopoeodi* Rogers, 1967	*Opsopoeodus emiliae* Hay, 1881	Leuciscidae	Pogonichthyinae	Mississippi	OM108533	OM108569
*D. ornatus* Rogers, 1967	*Notropis petersoni* Fowler, 1942	Leuciscidae	Pogonichthyinae	Mississippi	OM108534	OM108570
*D.* cf. *parvicirrus* Seamster, 1948	*Notemigonus crysoleucas* (Mitchill, 1814)	Leuciscidae	Leuciscinae	New York	OM108527	OM108563
*D. pectenatus* Mayes, 1977	*Pimephales promelas* Rafinesque, 1820	Leuciscidae	Pogonichthyinae	Wisconsin	OM108535	OM108571
*D. perlus* Mueller, 1938	*Luxilus chrysocephalus*	Leuciscidae	Pogonichthyinae	Arkansas	OM108536	OM108572
*D. rhinichthius* Wood & Mizelle, 1957	*Rhinichthys atratulus*	Leuciscidae	Pogonichthyinae	Wisconsin	OM108537	OM108573
*D. semotilus* Wood & Mizelle, 1957 (variant A)	*Luxilus chrysocephalus*	Leuciscidae	Pogonichthyinae	Arkansas	OM108538	OM108574
*D. semotilus* Wood & Mizelle, 1957 (variant B)	*Luxilus* sp.	Leuciscidae	Pogonichthyinae	Wisconsin	OM108539	OM108575
*D.* cf. *simplexus* Monaco & Mizelle, 1955	*Pimephales notatus*	Leuciscidae	Pogonichthyinae	Arkansas	OM108528	OM108564
*D. venusti* Rogers, 1967	*Cyprinella venusta* Girard, 1856	Leuciscidae	Pogonichthyinae	Mississippi	OM108552	OM108588
*Dactylogyrus* sp. 1	*Luxilus chrysocephalus*	Leuciscidae	Pogonichthyinae	Arkansas	OM108540	OM108576
*Dactylogyrus* sp. 2 variant A	*Cyprinella whipplei* Girard, 1856	Leuciscidae	Pogonichthyinae	Arkansas	OM108541	OM108577
*Dactylogyrus* sp. 2 variant B	*Cyprinella venusta*	Leuciscidae	Pogonichthyinae	Mississippi	OM108542	OM108578
*Dactylogyrus* sp. 3	*Luxilus chrysocephalus*	Leuciscidae	Pogonichthyinae	Arkansas	OM108543	OM108579
*Dactylogyrus* sp. 4	*Campostoma spadiceum* (Girard, 1856)	Leuciscidae	Pogonichthyinae	Arkansas	OM108544	OM108580
*Dactylogyrus* sp. 5	*Hypentelium nigricans* (Lesueur, 1817)	Catostomidae	Catostominae	Arkansas	OM108545	OM108581
*Dactylogyrus* sp. 6	*Hypentelium nigricans*	Catostomidae	Catostominae	Arkansas	OM108546	OM108582
*Dactylogyrus* sp. 7	*Cyprinella venusta*	Leuciscidae	Pogonichthyinae	Mississippi	OM108547	OM108583
*Dactylogyrus* sp. 8	*Catostomus commersonii* (Lacepède, 1803)	Catostomidae	Catostominae	New York	OM108548	OM108584
*Dactylogyrus* sp. 9	*Rhinichthys cataractae* (Valenciennes, 1842)	Leuciscidae	Pogonichthyinae	New York	OM108549	OM108585
*Dactylogyrus* sp. 10	*Rhinichthys cataractae*	Leuciscidae	Pogonichthyinae	New York	OM108550	OM108586
*Dactylogyrus* sp. 11	*Chrosomus neogaeus*	Leuciscidae	Laviniinae	Wisconsin	OM108551	OM108587

## Data Availability

Parasite voucher material is available at the Parasitology Laboratory, Department of Botany and Zoology, Faculty of Science, Masaryk University. DNA sequence data are available in the GenBank Nucleotide Database under accession numbers OM108517-OM108588.
